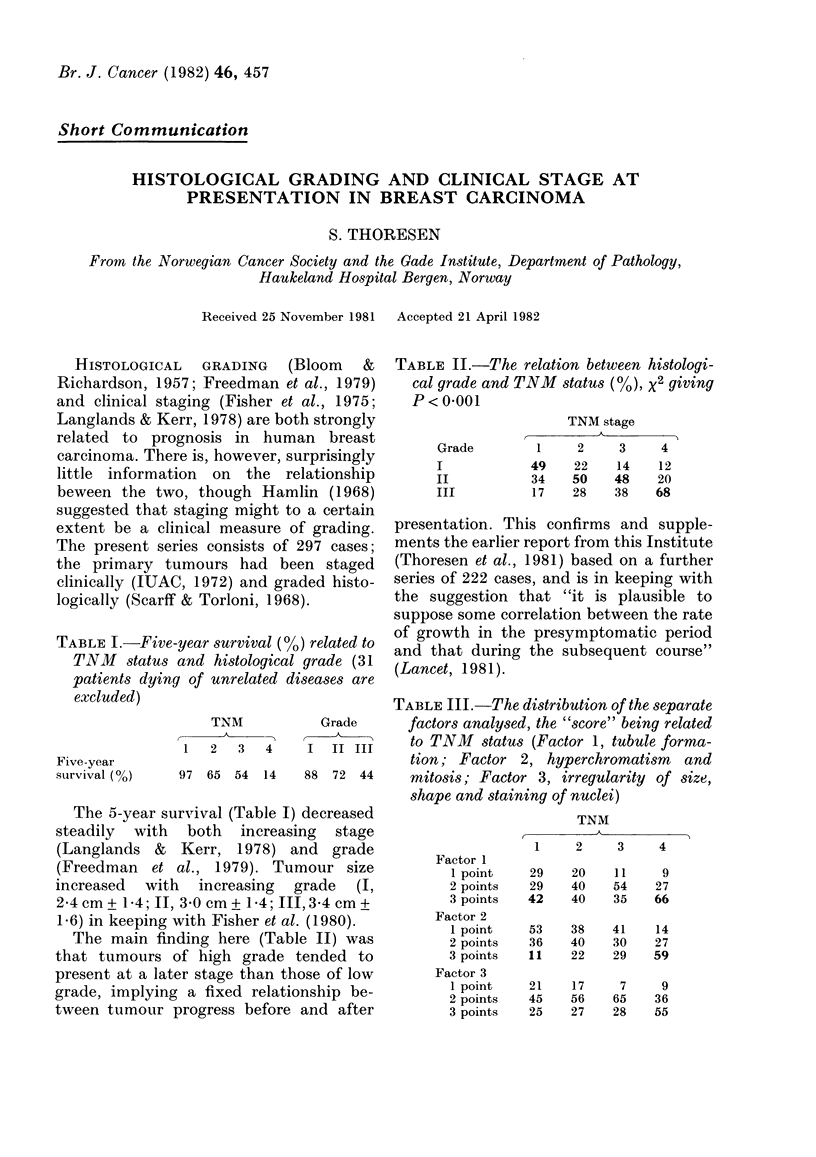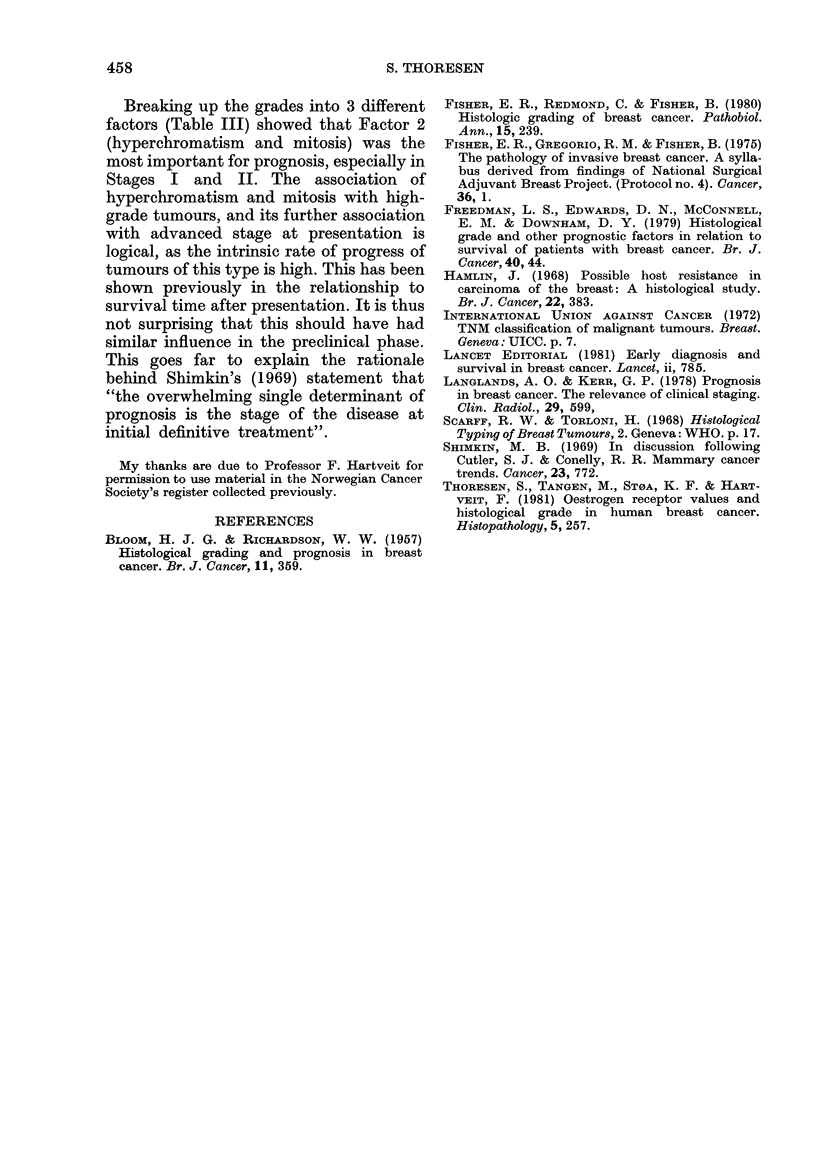# Histological grading and clinical stage at presentation in breast carcinoma.

**DOI:** 10.1038/bjc.1982.226

**Published:** 1982-09

**Authors:** S. Thoresen


Br. J. Cancer (1982) **46**, 457

*Short Communication*

### HISTOLOGICAL GRADING AND CLINICAL STAGE AT PRESENTATION IN BREAST CARCINOMA

S. THORESEN

*From the Norwegian Cancer Society and the Gade Institute, Department of Pathology, Haukeland Hospital Bergen, Norway*

Received 25 November 1981 Accepted 21 April 1982

HISTOLOGICAL GRADING (Bloom & Richardson, 1957; Freedman *et al.*, 1979) and clinical staging (Fisher *et al.*, 1975; Langlands & Kerr, 1978) are both strongly related to prognosis in human breast carcinoma. There is, however, surprisingly little information on the relationship beween the two, though Hamlin (1968) suggested that staging might to a certain extent be a clinical measure of grading. The present series consists of 297 cases; the primary tumours had been staged clinically (IUAC, 1972) and graded histologically (Scarff & Torloni, 1968).

TABLE I.—*Five-year survival (%) related to TNM status and histological grade (31 patients dying of unrelated diseases are excluded)*

| | TNM | | | | Grade | | |
|---|---|---|---|---|---|---|---|
| | 1 | 2 | 3 | 4 | I | II | III |
| Five-year survival (%) | 97 | 65 | 54 | 14 | 88 | 72 | 44 |

The 5-year survival (Table I) decreased steadily with both increasing stage (Langlands & Kerr, 1978) and grade (Freedman *et al.*, 1979). Tumour size increased with increasing grade (I, 2·4 cm ± 1·4; II, 3·0 cm ± 1·4; III, 3·4 cm ± 1·6) in keeping with Fisher *et al.* (1980).

The main finding here (Table II) was that tumours of high grade tended to present at a later stage than those of low grade, implying a fixed relationship between tumour progress before and after presentation. This confirms and supplements the earlier report from this Institute (Thoresen *et al.*, 1981) based on a further series of 222 cases, and is in keeping with the suggestion that "it is plausible to suppose some correlation between the rate of growth in the presymptomatic period and that during the subsequent course" (*Lancet*, 1981).

TABLE II.—*The relation between histological grade and TNM status (%), $\chi^2$ giving $P < 0{\cdot}001$*

| | TNM stage | | | |
|---|---|---|---|---|
| Grade | 1 | 2 | 3 | 4 |
| I | **49** | 22 | 14 | 12 |
| II | 34 | **50** | **48** | 20 |
| III | 17 | 28 | 38 | **68** |

TABLE III.—*The distribution of the separate factors analysed, the "score" being related to TNM status (Factor 1, tubule formation; Factor 2, hyperchromatism and mitosis; Factor 3, irregularity of size, shape and staining of nuclei)*

| | TNM | | | |
|---|---|---|---|---|
| | 1 | 2 | 3 | 4 |
| Factor 1 | | | | |
| 1 point | 29 | 20 | 11 | 9 |
| 2 points | 29 | 40 | 54 | 27 |
| 3 points | **42** | 40 | 35 | **66** |
| Factor 2 | | | | |
| 1 point | 53 | 38 | 41 | 14 |
| 2 points | 36 | 40 | 30 | 27 |
| 3 points | **11** | 22 | 29 | **59** |
| Factor 3 | | | | |
| 1 point | 21 | 17 | 7 | 9 |
| 2 points | 45 | 56 | 65 | 36 |
| 3 points | 25 | 27 | 28 | 55 |

Breaking up the grades into 3 different factors (Table III) showed that Factor 2 (hyperchromatism and mitosis) was the most important for prognosis, especially in Stages I and II. The association of hyperchromatism and mitosis with high-grade tumours, and its further association with advanced stage at presentation is logical, as the intrinsic rate of progress of tumours of this type is high. This has been shown previously in the relationship to survival time after presentation. It is thus not surprising that this should have had similar influence in the preclinical phase. This goes far to explain the rationale behind Shimkin's (1969) statement that "the overwhelming single determinant of prognosis is the stage of the disease at initial definitive treatment".

My thanks are due to Professor F. Hartveit for permission to use material in the Norwegian Cancer Society's register collected previously.

### REFERENCES

Bloom, H. J. G. & Richardson, W. W. (1957) Histological grading and prognosis in breast cancer. *Br. J. Cancer*, **11**, 359.

Fisher, E. R., Redmond, C. & Fisher, B. (1980) Histologic grading of breast cancer. *Pathobiol. Ann.*, **15**, 239.

Fisher, E. R., Gregorio, R. M. & Fisher, B. (1975) The pathology of invasive breast cancer. A syllabus derived from findings of National Surgical Adjuvant Breast Project. (Protocol no. 4). *Cancer*, **36**, 1.

Freedman, L. S., Edwards, D. N., McConnell, E. M. & Downham, D. Y. (1979) Histological grade and other prognostic factors in relation to survival of patients with breast cancer. *Br. J. Cancer*, **40**, 44.

Hamlin, J. (1968) Possible host resistance in carcinoma of the breast: A histological study. *Br. J. Cancer*, **22**, 383.

International Union against Cancer (1972) TNM classification of malignant tumours. *Breast. Geneva:* UICC. p. 7.

Lancet Editorial (1981) Early diagnosis and survival in breast cancer. *Lancet*, ii, 785.

Langlands, A. O. & Kerr, G. P. (1978) Prognosis in breast cancer. The relevance of clinical staging. *Clin. Radiol.*, **29**, 599,

Scarff, R. W. & Torloni, H. (1968) *Histological Typing of Breast Tumours*, 2. Geneva: WHO. p. 17.

Shimkin, M. B. (1969) In discussion following Cutler, S. J. & Conelly, R. R. Mammary cancer trends. *Cancer*, **23**, 772.

Thoresen, S., Tangen, M., Støa, K. F. & Hartveit, F. (1981) Oestrogen receptor values and histological grade in human breast cancer. *Histopathology*, **5**, 257.